# Exploring Community Psychosocial Stress Related to Per- and Poly-Fluoroalkyl Substances (PFAS) Contamination: Lessons Learned from a Qualitative Study

**DOI:** 10.3390/ijerph17238706

**Published:** 2020-11-24

**Authors:** Eric E. Calloway, Alethea L. Chiappone, Harrison J. Schmitt, Daniel Sullivan, Ben Gerhardstein, Pamela G. Tucker, Jamie Rayman, Amy L. Yaroch

**Affiliations:** 1 Gretchen Swanson Center for Nutrition, Omaha, NE 68114, USA; achiappone@centerfornutrition.org (A.L.C.); ayaroch@centerfornutrition.org (A.L.Y.); 2 College of Public Health, University of Nebraska Medical Center, Omaha, NE 68198, USA; 3 Department of Psychology, University of Arizona, Tucson, AZ 85721, USA; hschmitt@email.arizona.edu (H.J.S.); swolf22@arizona.edu (D.S.); 4 Agency for Toxic Substances and Disease Registry, Atlanta, GA 30341, USA; fty9@cdc.gov (B.G.); pgt0@cdc.gov (P.G.T.); fpe7@cdc.gov (J.R.)

**Keywords:** environmental contamination, mental health, psychosocial stress, stress coping capacity, public health response, community engagement

## Abstract

The purpose of this study was to qualitatively explore the per- and poly-fluoroalkyl substances (PFAS) exposure experience and associated stressors, to inform public health efforts to support psychosocial health and resilience in affected communities. Semi-structured interviews (*n* = 9) were conducted from July–September 2019 with community members and state public health department representatives from areas with PFAS-contaminated drinking water. Thematic analysis was completed and themes were described and summarized. Reported stressors included health concerns and uncertainty, institutional delegitimization and associated distrust, and financial burdens. Interviewees provided several strategies to reduce stress and promote stress coping capacity and resilience, including showing empathy and validating the normalcy of experiencing stress; building trust through visible action and sustained community engagement; providing information and actionable guidance; discussing stress carefully; fostering stress coping capacity and resilience with opportunities to build social capital and restore agency; and building capacity among government agencies and health care providers to address psychosocial stress. While communities affected by PFAS contamination will face unavoidable stressors, positive interactions with government responders and health care providers may help reduce negative stress. More research on how best to integrate community psychosocial health and stress coping and resilience concepts into the public health response to environmental contamination could be helpful in addressing these stressors.

## 1. Introduction

Experiences of chronic environmental contamination—such as learning that one’s drinking water has been contaminated with chemicals from industrial activity—impact millions of U.S. residents [1,2]. In addition to posing exposure-related physiological health risks, environmental contamination events can also affect psychosocial health, including stress, anxiety, and depression [2]. The National Academies of Science recommended examining psychosocial stress (hereinafter referred to as “stress”) as a potential risk modifier when studying environmental health risks [3]. Elevated stress may interfere with functioning of protective toxicokinetic processes, thus impairing an individual’s ability to recover from toxic exposures [4,5,6]. Further, stress has been shown to contribute to and modify health effects such as cardiovascular issues [7,8,9], inflammatory response [10,11,12], and immune response [13,14]. Therefore, stress may exacerbate health impacts that result from environmental contamination exposure.

Evidence suggests that psychosocial effects of environmental contamination vary across communities and community members, with some, but not all, people experiencing stress, anxiety, and/or depression, among other effects [15]. Those at increased risk for stress and worry related to experiencing environmental contamination include racial/ethnic minorities, women, people from low-income households, and people with disabilities [16,17,18,19,20]. A recent review [21] indicated that experiencing chronic environmental contamination presents a modest but robust overall effect on increasing general stress and anxiety, depression, and post-traumatic stress disorder symptoms.

Contamination-impacted community members may face many stressors. Examples of stressors include pervasive uncertainty (e.g., related to health risks and/or the contamination clean-up process); management of health problems and worry about future health; long duration impacts on day-to-day activities (e.g., water sources and usage); and complex chronic social stressors (e.g., social fragmentation and institutional delegitimization) involving community members and/or government agencies [22,23,24,25,26,27]. Additionally, psychosocial health implications and impacts (e.g., degrees of stress and relevant sources of stress) can vary across different contaminants [28]. Further, contamination-induced stress can aggravate existing socioeconomic and interpersonal stressors, as disadvantaged social groups (e.g., racial/ethnic minority and lower income communities) are more likely to be impacted by environmental contamination [4,25]. How an individual perceives and reacts to environmental contamination can be conceptualized as their “exposure experience” [29]. Understanding how community and/or governmental responses influence exposure experiences and stress can help inform a more effective public health and community-centered response.

The specific type of contamination event may influence the stress profile of an exposure experience. Environmental contamination can present very differently, such as natural versus technological, acute or chronic, and accidental or deliberate. Per- and poly-fluoroalkyl substances (PFAS) are a class of contaminants of concern in the United States and globally. PFAS include over 4000 chemicals manufactured for various commercial purposes, such as use in non-stick food contact surfaces, water-repellant fabrics, and in firefighting aqueous film-forming foams [30,31]. PFAS are persistent in the environment [32]. Humans are exposed largely through contaminated drinking water, contaminated food sources, and food contact surfaces [33]. Although PFAS are widely distributed across the environment due to their persistent nature, particularly high levels of localized PFAS contamination are associated with facilities that manufacture or use PFAS products such as manufacturing plants, waste processing facilities, dumpsites for PFAS products, airfields, military installations, and fire fighter training areas [33]. The direct health impacts of PFAS exposure is an emerging area of research, but early evidence suggests some PFAS have a long serum half-life and exposure may be associated with numerous adverse health impacts including reproductive issues, adverse immune responses, dyslipidemia, endocrine disruption, increase risk of some cancers, and effects on growth, learning, and behavior of infants and older children [33,34].

While there is a growing body of research on the health effects of PFAS, the exposure experience and related psychosocial health implications have been less well documented. PFAS contamination events are human-caused, pose uncertain health risks, often have long periods from first exposure to discovery, and may last for decades or longer until remediated. Qualitative methods allow for rich exploration of this understudied area [35]. This study qualitatively explored the PFAS exposure experience and associated stressors to inform public health efforts to address psychosocial health in affected communities.

## 2. Materials and Methods

### 2.1. Overview

We conducted nine semi-structured interviews from July to September 2019 with community members from PFAS-affected communities (*n* = 6) and state public health department representatives (*n* = 3) with PFAS-contamination experience. This work was conducted as part of the Agency for Toxic Substances and Disease Registry’s (ATSDR’s) Community Stress and Resilience Project [36]. The ultimate goal of these interviews was to inform development of materials, resources, and strategies for addressing community stress as part of the public health response to contamination. Given the lack of scholarship specific to this topic, these in-depth interviews offer rich initial findings and recommendations for public health practice and future research. However, due to the small sample size and limited perspectives of the interviewees, these findings are exploratory in nature. This study was conducted in accordance with prevailing ethical standards and protocols were reviewed and approved by the University of Nebraska Medical Center’s Institutional Review Board.

### 2.2. Sampling and Perspective

ATSDR identified prospective interviewees and invited them (through phone calls and/or email) to participate in an interview. This purposeful sample was selected due to their perceived ability to provide information relevant to experiencing PFAS contamination and related psychosocial health implications (e.g., based on professional background, involvement in PFAS advocacy efforts and conferences, and/or participation in stress-focused projects and activities) [37,38]. We limited the sample to nine individuals. This was due to pragmatic and administrative constraints [39] related to the timeline and information needs of ATSDR’s Community Stress and Resilience Project. 

Community member interviewees lived in areas with recent PFAS drinking water contamination and were involved in grassroots efforts focused on PFAS. Similarly, state public health department representatives had been involved in community outreach efforts as part of the public health response to localized PFAS contamination issues. We sought a diverse sample based on geography, source of local PFAS pollution, professional background (e.g., health-related vs. not), and by ensuring people of color were included. Once interviewees agreed, an ATSDR subcontractor scheduled and conducted the interviewees. 

### 2.3. Procedure

Semi-structured 60 min interviews were conducted by telephone. Telephone interviews, compared to in-person interviews, are more feasible to conduct with a small sample spread out over a large geographic space. Two of the non-ATSDR study authors were in attendance for each interview, with one leading the interview and the other taking notes. The interviews were audio recorded and transcribed with participants’ permission (one interviewee opted to only have detailed notes taken).

The authors drafted the interview guide collaboratively. Table 1 outlines the core interview questions asked in the interviews of the community members and public health department representatives.

The qualitative approach incorporated thematic analysis utilizing Creswell’s “lean coding” technique [40]. Themes were allowed to emerge inductively from all sections of the interviews. Researchers met regularly to discuss preliminary findings and noted emerging themes, meanings, and relationships among themes. A coding guide was created in three iterative steps. First, two researchers both read and open-coded two randomly selected interviews to begin to understand the data and develop categories [41]. During open coding, researchers read the transcripts and made memos concerning meaningful aspects of the interviewees’ experiences, such as activities, events, interactions, stressors, lessons learned, and recommendations. No predefined codes or categories were used. Next, researchers met to discuss the open coding to build a list of codes for themes and sub-themes. Finally, the research team began coding the transcripts using NVivo qualitative analysis software, and met frequently to discuss the adequacy of the coding list, revising as necessary (e.g., combining codes when duplication/overlap became apparent, removing codes that were not used frequently, and grouping/re-grouping codes and sub-codes when needed). Illustrative quotes from the interviews are provided throughout the findings section. Quotes are coded as Community Member (CM) or Health Department (HD) interviewee and numbered. 

Given the focused nature of the interviews (e.g., actions, perceptions, stress and coping, and recommendations) and similar perspectives of the interviewees, thematic saturation was reached quickly. Additionally, comparison of emergent themes to findings from recent quantitative and qualitative PFAS-related studies [35,42,43] added assurance that thematic saturation was reached. While no new themes related to the study objectives emerged during the latter interviews, additional sampling could have allowed us to more fully explore themes (e.g., add context, description, and examples). However, due to external constraints mentioned earlier, further sampling was not possible. 

Member checking occurred following analysis. Interviewees were given the opportunity to review an initial draft of the results and synthesis table to validate that the findings and conclusions aligned with their own experiences with PFAS contamination. Three interviewees provided feedback, one community member and two health department interviewees. Interviewees agreed with the findings and recommendations provided. They also noted opportunities to add detail and clarify their quotes in the manuscript.

## 3. Results

### 3.1. Sample Characteristics

The six community member interviewees’ current or former professions and roles in their community included education, healthcare/mental health, social work, politics/government, military, non-profit organizations, and business. The three state public health department representative interviewees all participated in site-specific PFAS responses and had experience in community outreach/engagement, public communication, health education, and/or risk assessment. The nine interviewees included seven women and two men, representing six states (Alabama, Arizona, Colorado, Michigan, New Hampshire, and North Carolina). All six community members resided in different states, and all three state public health department representatives worked in different states. For general demographic characteristics, the six cities that community member interviewees lived in included greater than national average percentages of African Americans (*n* = 1), Caucasians (*n* = 2), Hispanics/Latinos/as (*n* = 1), and two cities whose racial/ethnic distribution was similar to national averages. Additionally, two of the cities had poverty rates above 20%, two had poverty rates between 10–19%, and two had poverty rates below 10%.

### 3.2. PFAS Contamination and Discovery

The sources of PFAS as reported by community member interviewees included both industrial activity (four communities) and military-base/non-military airport (two communities). All the communities represented by the community member interviewees had multiple locations where contamination had been detected in drinking water, with at least one in each community being greater than the Environmental Protection Agency’s Health Advisory level for perfluorooctanoic acid (PFOA) and perfluorooctanesulfonic acid (PFOS) in drinking water (i.e., ≥70 parts per trillion (ppt) (0.07 μg/L), individually or combined). The Health Advisory does not represent a definitive cut-off between safe or unsafe conditions, but rather provides a margin of protection for individuals throughout their life from possible adverse health effects. Further, most communities had found other forms of PFAS (in addition to PFOA and PFOS) in their drinking water. Community member interviewees reported first learning about local PFAS contamination within the past two to six years (2013–2017). Although it is not known exactly how long the communities had been contaminated with PFAS, the activities that likely caused the contamination had been ongoing for many years prior to the community members’ discovery of contamination. Community member interviewees became aware of the contamination from local news stories, through word-of-mouth, and via mailed or in-person communication with governmental officials (e.g., an agency was conducting door-to-door water tap testing). 

### 3.3. Community Member Actions and Interactions with Government and Health Care Providers

Community member interviewees described that when they initially learned about PFAS contamination there was little information immediately available, so many sought information on the internet. Additionally, other early sources of PFAS information mentioned included paper and digital materials provided by governmental agencies and water authorities. Nearly all community member interviewees mentioned forming local community groups in response to learning about contamination. Key functions of these groups included disseminating information, advocating for PFAS-focused public health actions, and collaborating with experts and other organizations (i.e., researchers, toxicologists, medical professionals, non-profits, universities, water companies). 

Community member interviewees primarily interacted with governmental agencies about PFAS contamination. Perceptions of government response to PFAS were mixed. Some community member interviewees described that their public health departments and environmental agencies were proactive in testing water and establishing drinking water advisories, while others perceived that these agencies were slow to respond and/or did not respond appropriately (e.g., setting relatively high drinking water advisory levels for PFAS; and/or working with polluters to reduce the amount of PFAS released in the environment, rather than stopping operations to prevent further releases). All community member interviewees reported at least some initial suspicion and/or skepticism about their governments’ actions (primarily local-level government, although state-level government was also mentioned in this context). 

Several interviewees described interactions with their health care providers concerning PFAS. They reported that many physicians were either unaware of the problem or lacked information on how to address medical concerns related to PFAS exposure. Some interviewees indicated that they had to educate their physicians about PFAS. Although interviewees viewed their physicians as trusted sources of information, they believed physicians were largely ill-equipped to respond to patients’ PFAS concerns. However, interviewees reported that some physicians took an active role in information sharing by giving presentations during community forums. One interviewee (who interacted regularly with several physicians as part of their community PFAS advocacy efforts) reported that once physicians in their community learned PFAS was an endocrine disrupter, the physicians were motivated to take PFAS and related health impacts more seriously. In another instance, a physician was the first to definitively advise community members not to drink the PFAS-contaminated water. The interviewee said they appreciated this clear advice and firm stance. 

### 3.4. Sources of Stress Related to Experiencing Community PFAS Contamination

When asked about the stressors associated with community experience of PFAS contamination, community member interviewees brought up several common concerns: health issues, loss of trust in governmental institutions, and financial burdens incurred due to PFAS contamination. A fundamental aspect of these stressors among community member interviewees was the pervasive sense of uncertainty, frustration, and lack of control over the situation.

#### 3.4.1. Health Concerns and Uncertainty

All interviewees discussed concerns about the prevalence of health issues in their communities, especially various cancers, kidney issues, and fertility/reproductive issues. Stories about health issues came up throughout the community member interviews, reflecting the important role of health concerns in contributing to the stress experience of PFAS contamination. Community members’ concerns over health issues frequently extended to deaths, especially unexpected deaths (i.e., children or seemingly healthy individuals). One community member described unexpected deaths witnessed in their community, *“We’re actually devastated because there’s so much death around us … All these deaths are unexpected. We are just trying to cope with it …”* (CM5). Interviewees’ also expressed health concerns and uncertainties about potential various PFAS exposure routes, including soil and water in washing machines, showers, recreational swimming areas, and irrigation systems for food production. In addition, community interviewees expressed consternation over the lack of access to blood tests to determine how much PFAS they and their families had been exposed to. Health concerns appear to result in a fundamental uncertainty about who would be affected, when they would be affected, and how they would be affected. 

While all interviewees mentioned health concerns as a major contributor to stress, interviewees differed on the role of PFAS and appropriate next steps to take. Community member interviewees conveyed more certainty that PFAS had caused health issues within their communities, but were uncertain about how and when PFAS contamination would affect them and their family members. Alternatively, health department interviewees expressed greater uncertainty about the science linking PFAS exposure and health issues, and conveyed ambiguity around whether actions such as biomonitoring (i.e., testing community members’ serum to assess levels of PFAS) would help the community in a meaningful way. One health department interviewee described the differing perspectives: *“(There needs to be) recognition that we don’t know what exposure means for someone’s health … There was a general concern amongst people that their health conditions were connected to PFAS … even if the science doesn’t support that assumption.”* (HD3).

#### 3.4.2. Institutional Delegitimization and Loss of Trust

Other major stressors discussed by interviewees in the context of PFAS contamination were institutional delegitimization—feeling that one’s health concerns and other aspects of the contamination experience were being delegitimized by relevant institutions—and the loss of trust in governmental and health agencies previously seen as “protectors.” Instances where government or health officials downplayed the severity of the issue of contamination were reported as sources of stress and frustration for community member interviewees. An interviewee described the perception of governmental officials minimizing community members’ concerns, *“What we were hearing was ‘there is nothing to see here. You guys are being hysterical. There’s nothing to worry about’.”* (CM4). By failing to validate that feeling concerned, anxious, stressed, and/or angry (all of which were emotions expressed by interviewees related to these experiences) is a natural or understandable reaction to the situation, community member interviewees reported that officials delegitimized their lived experience. For example, this reportedly occurred when health officials used equivocal language about health effects in response to community member concerns, citing the lack of a definitive scientific link between PFAS and health issues. Similarly, perceived governmental inaction (e.g., failure to take punitive action or enact regulations on polluters) and hesitancy to assign blame contributed to feelings of delegitimization and loss of trust. Further, perceptions that government officials were collaborating with the industries responsible for PFAS contamination, contributed to community member interviewees losing trust in government. 

#### 3.4.3. Financial Burdens

Interviewees discussed the financial burden resulting from PFAS contamination as another stressor. After learning about PFAS drinking water contamination, many people sought ways to reduce exposure by purchasing and installing water filtration systems or drinking bottled water. Both community member and health department interviewees expressed concerns that community members may take on financial burden to reduce exposure. Concerns about healthcare access and the high cost of blood tests for PFAS levels were also significant financial stressors discussed by community member interviewees. Interviewees expressed concern that the discovery of PFAS contamination has led to declining property values, inability to sell houses, and lost income for rural farmers with contaminated soil. One health department interviewee provided an example of the impact PFAS stigma can have on property values: *“I talked to a couple in [CITY NAME REDACTED] when they were trying to sell their house. They lived across the road from the investigation area and they wanted to move where their kids lived. They couldn’t sell their house because now it has the stigma that it’s in this contaminated zone and they end[ed] up just leaving, they just left their house because they wanted to be with their kids.”* (HD2).

### 3.5. Advice for Stress-Reducing Public Health Response

Community members and health department interviewees were asked to provide their advice for governmental agencies responding to communities dealing with PFAS contamination, especially advice for reducing stress and/or avoiding making a stressful situation worse. See summary in Table 2 (end of this section).

#### 3.5.1. Empathy, Validation, and Trust

Interviewees emphasized the importance of governmental representatives practicing empathy as a fundamental aspect of community interactions. One public health department representative with extensive community engagement experience stated: *“That doesn’t maybe get done enough—is just to sit and listen and empathize with people and the situation that they’re going through”* (HD1). Validating the legitimacy of health concerns and fears (even if the science is not conclusive), rather than dismissing or minimizing community concern, was discussed as a crucial component of empathetic interactions. One community member described their experience interacting with local health department personnel as an attempt to minimize community concern, *“they start out (by saying) … ‘They measure parts per trillion. That’s one drop in an Olympic-sized swimming pool. I mean how bad can that be?’ … I think they think they’re being reassuring to people and I think what people feel like is that’s being dismissive of their fears”* (CM4). Related to validating concern, community member interviewees also conveyed a sense that their observations of health impacts were being dismissed because they were not experts. One community member described how lay knowledge was dismissed in their experiences interacting with governmental officials, *“… people know what they know. And they can connect the dots, but if you don’t have the official folks behind you, and feel validated-then it’s considered anecdotal.”* (CM2).

Health department interviewees described striking a delicate balance when interacting with the community. They were concerned that their comments could be misconstrued as official statements about health risks. One health department interviewee summed up this point as, *“… we’re a big bureaucracy and there are people who can speak to certain issues and there are people who can’t speak to certain issues”* (HD2). Health department interviewees reported a hesitancy to stray far from well-established scientific facts when interacting with the public. One health department interviewee described this barrier to open communication as, *“That tends to be the comfortable spot that we speak from … We tend to speak from a standpoint of science. Whereas what people in the community need is to be listened to and empathized with; to feel like they’re being heard, and their concerns are being addressed”* (HD3).

Interviewees conveyed that while empathy and validation is crucial, it should also be followed by governmental actions that would re-establish trust with affected communities. Interviewees explained that governmental representatives need to demonstrate respect by being honest and humble in how they interact with the community. This includes acknowledging that community members were wronged and avoiding condescension in verbal interactions, as well as implausible or disingenuous claims. Further, interviewees advised that governments should not only foster a two-way dialogue between themselves and the community, but also facilitate communication between community members through forums and meetings. One community member described their negative impressions of a community PFAS forum they attended, *“So they sat 11 people on the stage … which is a higher plane than everybody else. It was perceived that they are sitting higher than us, looking down on us, giving us information, and some of them literally had smirks on their faces. And when the community was upset, they had a person managing the conversation say, ‘oh, well, just don’t be so angry’.”* (CM1). Interviewees reported preferring a straightforward, honest, and open approach to discussing PFAS and related issues. On scientifically complex topics, interviewees advised assuming audience intelligence, but also providing tools (e.g., cheat sheets or fact sheets) and explanation for those who might need additional context. Additionally, interviewees welcomed the use of visuals and plain language text to help explain complex topics (e.g., ensuring information is not only communicated verbally, but also visually when giving presentations at community forums). One interviewee said, *“… a lot of folks are visual. They can see what you’re talking about. A lot of folks can read what you’re talking about. And the comprehension piece comes into place.”* (CM6).

For health department interviewees, ongoing information provision and effective risk communication were described as a key for building trust with their community. One interviewee stated: *“… it goes back to that ‘be first, be right’ risk communication and establishing from the get-go that relationship with the community so that when there is this misinformation, they trust you to correct it”* (HD1). Further, this interviewee went on to emphasize the importance of not only establishing a trusting relationship with the community, but also nurturing that relationship through continued follow-up and visible actions to address the PFAS situation: *“Really the only way we can counter [lack of trust with the government] is through actions and continued communication … There are people in our state who definitely still do not trust us. One of the best things you can do as government agencies is just to continue to be in contact with the community, to continue to show them that we’re engaged, and that we’re here and we’re doing everything we can.”* (HD1).

One community member illustrated the importance of frankness and providing actionable guidance to re-establish trust, advising: *“Government should be honest and realistic when speaking with the community. Do not try to mitigate fear, but instead be up front about what you don’t know and what you can’t officially say. But then offer practical and realistic guidance that is based in caution around chemical exposure, rather than leaning on the fact that ‘health impacts aren’t yet proven’.”* (CM3).

#### 3.5.2. Information Dissemination to the Public

Interviewees indicated that one of the main functions of public health departments is to inform the public. As reported by community member interviewees, the uncertainty surrounding PFAS was a major source of anxiety and fear. Community member interviewees reported wanting information on PFAS facts, health risks, safe levels, interpreting testing results, actions to reduce exposure, and medical treatments. Providing as much credible information and actionable guidance as possible shortly after the discovery of PFAS contamination may help reduce stress related to uncertainty. One interviewee said, *“When you have knowledge, you become less fearful” (CM4)*. However, as noted by health department interviewees, some of the information community members wanted was not available at the time and/or was evolving (e.g., safe levels for drinking water).

Interviewees described many communication channels for disseminating information about PFAS contamination to the public. All interviewees described a traditional town hall or community forum approach being at least one aspect of the information dissemination strategy—sometimes government-led and other times community-led. However, interviewees also emphasized the importance of one-on-one connections. Large forums were described as the best approach to “push out” information as they ensured that everyone attending receives the same information. One-on-one interactions were recommended for showing empathy and establishing trust. Two interviewees (one community member and one health department interviewee) discussed integrating the two approaches, i.e., using a “health fair” configuration—that allows governmental agencies, health care providers, and community groups to assemble tables to connect one-on-one with community members for the first hour, followed by a group forum for presenting information in the second hour. Interviewees that implemented this approach reported it was well received by the community. Interviewees also suggested disseminating information through the local news, social media/websites, signs/bulletin boards, mail, door-to-door, and through local organizations. 

While such channels can reach a wide audience, they may not reach everyone. Interviewees highlighted the importance of an inclusive and broad communication approach that considers the needs of various “hard-to-reach” audiences, such as those that do not speak English, the home-bound elderly, and low-income populations who may not have internet access, or access to reliable transportation or schedule flexibility to attend meetings. Employing a communications planning taskforce that includes diverse perspectives was proposed by interviewees as a promising strategy to help ensure a broad reach. Further, interviewees emphasized understanding communication and information needs and preferences among low-income groups when providing guidance. It was reported that low-income populations may be last to get information or may not be able to act on advice due to economic constraints. One community member recalled guidance given in their community to use in-home certified PFOS/PFOA water filters. However, there was no financial support provided for obtaining such filters so low-income families *“… often had to continue to use the water even though they know it is bad”* (CM3).

#### 3.5.3. Communicating Stress Risk

While all interviewees acknowledged the significance of the stress induced by experiencing PFAS contamination, few reported that the topic had been addressed directly in their communities, or, if it had, efforts were limited. One community member explained, *“There was nobody [at the community forum] that had the mental health background, and that has not been discussed at all in our community even now—the whole mental health piece of it. And we do have people that are really struggling—really struggling—with this”* (CM4). 

Health department interviewees reported that discussing stress in the context of PFAS contamination was a potentially contentious topic for the public. Community member interviewees conveyed that if trust was not established prior to discussing stress, there would likely be skepticism from the community and the message may “fall flat.” Further, interviewees warned that discussing stress could easily be interpreted as “victim blaming” or dismissive—that government agencies believe community members are “just stressed” or “over-reacting.” One health department interviewee discussed a failed attempt by their department to broach the topic of stress with the community: *“We have someone in public health who deals with community stress and anxiety … We brought that person early on to a community meeting and had her try and speak directly to the stress and anxiety people felt. The feedback we heard from that engagement was negative. The community felt talked down to and that their concerns were being minimized.”* (HD3). To reduce the chance of this, interviewees advised government agencies to discuss stress only after having empathized, established trust, and validated the community members’ physical health concerns. In short, first establish trust, and then frame stress within the context of other health concerns. 

#### 3.5.4. Building Social Capital and Restoring Agency

While interviewees suggested that the experience of PFAS contamination in a community can be stressful, there are some aspects of this experience that contribute to effective stress coping and positive outcomes. In particular, opportunities to meet others that were similarly affected can help build social capital. For example, community-led groups and forums allow community members to meet each other, form bonds, share stories, and validate each other’s experiences with contamination. These interactions can be helpful both within and between communities (e.g., the National PFAS Contamination Coalition). Further, participating in and leading community action (e.g., advocating for water treatment and remediation; helping those most affected; sharing information with the community; and engaging with public health officials, researchers, and media) made community member interviewees feel empowered—restoring a sense of agency (i.e., the sense of having the power and capability to produce an effect or exert influence). One community member shared their perspective on the influence of the social aspects of connecting with other affected community members: *“(Stories from) people across the country...who lost people, are struggling with harm, and are worried about their children are appalling. But then you look at that person and see how strong they are, they decided to take that energy and do something about it. Those people are all over the country and they are supporting people in their communities and people are seeing them. In a weird way it’s giving people hope … People are feeling validated that they’re counted and that they are seen, and that validation is very important to victims.”* (CM2).

Along with community solidarity and collective action, interviewees also had suggestions for what government officials, health care providers, and media, can do to validate and empower affected community members. Positive interactions with government officials and health agencies—such as inviting community members to voice their concerns, involving community members in decision-making processes, and officials simply showing up to community meetings—were all described as important experiences that validated and empowered the community. These interactions also offer opportunities to form social bonds across community sectors (e.g., between community members and governmental officials). Community member interviewees reported feeling validated when researchers and media outlets took interest in investigating contamination and felt empowered when they were involved in investigations. Finally, interviewees suggested that health officials can support the community members in their personal empowerment by providing information and making specific suggestions on how they can reduce exposure and get involved in efforts to address PFAS contamination. This offers the person a way to reestablish a sense of agency. A health department interviewee gave the following advice: *“People feel like something has happened to them that they didn’t have control over. Providing them with steps they can take to give some control back to them—whether that be call your state representatives or switch to bottled water to reduce your exposure. I think those types of things help people feel more empowered …”* (HD1).

In addition to building social capital and agency, some interviewees discussed helping community members cope with stress by providing access to mental health services (e.g., counseling). This approach was discussed primarily by the health department interviewees, although several community member interviewees also mentioned it. Health department interviewees indicated that they were in the early stages of widening access to mental health services by having clinical social workers available during community forums and/or working with local mental health counseling agencies to increase access to sliding-scale-fee services for those affected by PFAS contamination. 

#### 3.5.5. Public Health Guidance and Training Needs

Interviewees discussed training and guidance needs for health professionals working directly with affected community members-especially public health department personnel and health care providers. For public health department personnel, interviewees felt training and guidance was needed for engaging with outraged communities, communicating risk, and integrating mental health counseling services into the public health response. One health department interviewee described a training they attended, *“We had a training here called ‘emotions, outrage and public participation’. So, it was about how to work with communities that are angry. My biggest takeaway from that was ‘people are angry, it’s not directed at you personally. It’s the situation that they’re frustrated with. Be empathetic, listen’.”* (HD2). Further, health department interviewees mentioned the need for more Federal guidance on responding to PFAS, as each state handles its PFAS response differently. One interviewee said, *“I think (it was) important for the federal government, in this case ATSDR, to step in and take a lead role in coordinating a response and providing guidance. Not only guidance around blood testing, but also guidance for health care providers and public communication. This is a key role for organizations like the CDC, but early on in our response there wasn’t a lot of coordination, and states were creating differing messaging and guidance.”* (HD3). Interviewees suggested reaching out to health care providers in and near PFAS contaminated areas with information related to how PFAS affects health, treatment and prevention approaches, understanding the stress implications of experiencing contamination, and how to screen for those that may be most at risk for exposure and health effects. Interviewees recommended integrating health care provider education into community outreach plans.

## 4. Discussion

While communities affected by PFAS contamination will face unavoidable stressors, positive interactions between community members, government responders, and other parties (e.g., health care providers) may help reduce stress. Public health professionals who are interacting with community members can start by recognizing that public health actions can have secondary impact on stress in communities [22,44,45]. When engaging community members, government representatives and agencies may promote positive and stress-reducing interactions by showing empathy; re-establishing and/or building trust; providing information and actionable guidance; discussing stress carefully; supporting stress-coping activities; and building governmental capacity to address stress. Further, environmental justice implications should be considered. This may include ensuring socially marginalized groups receive clear information via culturally appropriate methods, and ensuring recommended actions are feasible for low socioeconomic status groups. Integrating these considerations into robust response efforts may help form more supportive and less stressful community-government relationships. 

Primary stressors among community members identified in these interviews included health concerns and associated uncertainty, institutional delegitimization of concerns and associated distrust, and financial burdens incurred—all contributing to feelings of frustration and powerlessness. The presence of both social and material stressors in environmental contamination and disaster contexts has been described elsewhere [44,46,47]. Further, community members in other studies of PFAS-contaminated communities [35,42,43] and communities experiencing other chronic environmental contamination [24,48,49,50,51,52,53,54] have noted a similar set of stressors and feelings of powerlessness. This indicates the PFAS contamination experience may be similar to chronic environmental contamination from other sources and substances (e.g., solvents in drinking water, lead in drinking water and soil). 

Both community member and public health department interviewees emphasized uncertainty about health effects as a key characteristic related to how PFAS contributes to stress. PFAS contamination, as compared to a relatively more understood contaminant such as lead, was unfamiliar to those affected and public health professionals, especially initially. Being knowledgeable about a contaminant is associated with less worry [20]. The unsettled science on PFAS health risks added to public health departments’ challenges in providing clear guidance on PFAS health effects, likely contributing to stress in these communities [55,56]. 

Interviewees in the current study discussed division between community members and government, particularly local government. This divide was evident in the differences in the threshold for evidence needed by community members (who often relied on personal experience, as well as scientific evidence) and health department interviewees (who needed strong scientific evidence, as well as administrative authority/clearance in some cases) to make a link between PFAS and health risks. Such a disconnect has been observed in other contamination contexts as well [16,54,57]. This disconnect in the perception of the validity of PFAS risk likely contributed to governmental officials reportedly attempting to calm the community members’ feelings, which contributed to the community perception of having their concerns minimized. As noted in this study and others, community members experience such actions as delegitimizing their concern, which can lead to perceptions of victim blaming [58,59]. Further, community members perceived that governmental officials had sometimes made unrealistic claims, implying that PFAS was safe, though some evidence indicated otherwise (e.g., *“They measure parts per trillion. That’s one drop in an Olympic-sized swimming pool. I mean how bad can that be?”*). Such claims can erode credibility and lead to distrust [60]. Empathetic, honest, and practical communication, with credible information, is noted in the literature as key to building trust [23,61] and interviewees in this study echoed these sentiments.

Interviewees reported limited direct discussion about stress related to PFAS contamination, but it was unclear from these data why that was. Environmental health-focused government staff may view stress issues as outside their purview or area of expertise and/or community members may fear stigmatization regarding mental health [62,63]. Research indicates that validating the experience of negative stress as a normal reaction to the situation may help reduce stigmatization [64]. However, it is also helpful for governmental agencies to first assess the community’s interest in broaching the topic of stress [65]. If community members are resistant to addressing stress, doing so may be perceived as victim blaming, which would be counterproductive. Interviewees in this study described prerequisites for successful government stress intervention: establish trust and discuss stress in the context of other health concerns to reinforce the validation of health concerns. Therefore, a viable strategy for stress interventions may be (1) building trust, (2) assessing the openness of community members to discuss stress; and (3) partner with community members to develop and implement practical stress interventions without inadvertently minimizing other concerns.

Interviewees in this study largely discussed building and/or drawing on social capital and restoring agency as primary means to help communities cope with negative stress. Aldrich (2012) [66] discussed the importance of ‘bonding’ and ‘bridging’ social capital. Bonding social capital includes social ties among community members, while bridging social capital includes ties between the community and other sectors such as government and healthcare. Strategies for building social capital might include informal outreach events and establishing support groups [64,65]. In this study, community members prominently described the bonding social capital they built among community members (e.g., establishing community groups) in response to experiencing PFAS contamination. Additionally, although attempts at building bridging social capital sometimes included mixed results (e.g., community member interviewees described both positive and negative interactions with governmental agencies and health care providers), the community groups in this study were able to work with various sectors (e.g., government agencies, researchers, policymakers, journalists, etc.) to advocate for action on PFAS. However, it is not clear from these data the extent to which marginalized groups were included in opportunities to build bonding and bridging social capital. Although interviewees did not provide specific recommendations, an approach similar to the diverse communications planning taskforce suggestion for information dissemination (discussed earlier in the results) may be useful in planning inclusive strategies for building social capital. Developing this bridging social capital by building trust and nurturing personal relationships between community members and local government may be key to implementing any inclusive and collaborative efforts to address PFAS contamination [23,67].

To counter the powerlessness felt by community members, interviewees in this study and others [23,61,68] advise restoring agency. Restoring agency can not only help with recovery from negative psychological effects [65,69,70], but can also help empower communities through collaborative efforts to seek long-term solutions [71]. In the current study, interviewees suggested forming community advisory groups to share civic power and decision-making. This approach may help restore agency while also leveraging practical knowledge within the community and applying it in collaborative decision-making [68,71,72]. Research has shown that local knowledge can be especially important for gathering environmental and exposure information and understanding community needs and resources [2,45,69,73].

Given the extended time-course of chronic environmental contamination and potential for re-traumatization, affected communities and governments may benefit from sustaining stress intervention efforts to foster long-term stress coping capacity and resilience [4,64,74,75,76]. A stress intervention framework developed by Sullivan et al. [44] addresses stress in the context of chronic environmental contamination and presents similar strategies to those described by interviewees in this study. The Sullivan et al. [44] model advised those implementing stress interventions in affected communities (e.g., public health professionals) to legitimize the stress experience, communicate risk effectively, build lasting relationships, be sensitive to the chronic nature of the trauma, form community groups, and facilitate informal outreach and support opportunities. Implicit in the Sullivan et al. [44], model (and similar approaches; e.g., Sandifer and Walker, 2018 [76]; Abramson et al., 2015 [77]), and discussed by interviewees in this study, is the need for training and capacity building among public health department personnel that addresses stress and implementation of stress coping and resilience strategies in communities affected by contamination.

Due to several study limitations, these findings should be interpreted with caution. First, this exploratory study had a small sample size and relatively narrow perspective. For example, the sample did not include the perspectives of those not active in community groups, those that opposed PFAS advocacy efforts, and those representing other sectors and government jurisdictions/agencies. Further, the unique stress experiences of marginalized demographic groups (e.g., racial/ethnic minority groups, low-income households, those with disabilities, etc.), and related environmental justice implications, were not well-explored in this study. Research shows these groups, due to a historical backdrop of disenfranchisement, may be more likely to experience environmental contamination, certain common stressors (e.g., loss of trust in the government), and unique stressors (e.g., racism) than other groups [16,17,18,19,20]. Future studies should employ culturally appropriate outreach approaches to reach marginalized communities and explore their unique experiences. Additionally, this study presents a single snapshot in time. As the literature suggests [44], the long time-course of chronic environmental contamination is a primary feature contributing to stress. Longitudinal studies may prove well suited to explore stressors and subsequent development of intervention strategies for addressing stress during different temporal phases of chronic environmental contamination. 

## 5. Conclusions

Based on semi-structured in-depth interviews with a small sample of community members and state health department representatives, this qualitative study explored the exposure experience and related stressors among communities with PFAS drinking water contamination. Based on this initial exploration, we present practical recommendations to address psychosocial health issues as part of the public health response to PFAS contamination. The exposure experience and related stressors were similar to those reported in other communities affected by chronic environmental contamination. Therefore, stress intervention approaches that have proven effective in other situations may be promising in the PFAS contamination context. Consistent with a larger body of literature on this topic, this study found that while experiencing environmental contamination is stressful, community-based stress intervention approaches are not well-integrated in public health responses. There is a need for more research on how best to integrate stress concepts and intervention strategies into the public health response to environmental contamination. 

Finally, this study raises questions that might prove useful avenues for future research. These potential research areas include delving deeper into emotional experiences related to PFAS contamination; examining the PFAS experience among those belonging to marginalized groups, identify unique characteristics of their experiences, and related implications for stress intervention; and investigating contextual factors and implications for stress intervention in the PFAS context, such as the role of timing of intervention, historical experiences with environmental contamination, and level of trust among the community for the responding agencies. Addressing these research questions will provide those developing stress intervention approaches with better information to effectively tailor solutions to populations’ needs.

## Figures and Tables

**Table 1:** Core interview questions asked during the 60 min semi-structured interviews for the community member and public health department interviewees.

Community Members	Public Health Department Representatives
When PFAS contamination became apparent, would you describe your initial actions, thoughts, and feelings during the first month or so?	From what you observed, how was the day-to-day routine or lifestyle of the community affected initially?
What were the initial actions, thoughts, and feelings of those in your community that you know well (e.g., friends, family, neighbors, co-workers, etc.)?	From your perspective, what were the initial feelings and reactions of the community members to the PFAS contamination, at least as far as you are aware?
Would you describe actions government agencies took and how your community reacted?	Would you describe the initial actions or response of your agency/organization?
Were there any people, institutions, or providers that were especially helpful for the community, or from whom you were expecting more help (for example government agencies, scientists, or businesses)?	Would you describe the actions community groups and other organizations took, initially, when they learned about the PFAS contamination?
Would you describe how you feel your community is coping with knowing there is PFAS contamination?	Would you describe any resources, services, or support that has been especially crucial to the community’s response to the PFAS contamination?
What, if anything, has been most helpful? What have been the main barriers that have made it difficult?	Would you describe any resources, services, or support utilized specifically to help affected community members cope emotionally with becoming aware of the PFAS contamination in their community?
What do you wish was available, but wasn’t? Could you describe how this would have helped?	What do you think about the overall response to the contamination—including governmental agencies, scientists and experts, utilities companies, and relevant businesses? Have these institutions successfully worked with the community?
Based on your experience, what recommendation(s) would you give to government agencies and organizations to better support community members affected by PFAS contamination?	Do you have examples of situations where institutional representatives performed especially effectively, or where perhaps they struggled?
	Based on your experience, what would be the main recommendation(s) you would give to other public health agencies to best support and assist community members affected by PFAS contamination?

**Table 2:** Interviewee recommendations for public health and other government agencies on addressing psychosocial stress in communities impacted by PFAS contamination.

Recommendation	Description	Illustrative Quote
Show Empathy	Show empathy towards community members experiencing PFAS contamination, listen and understand their concerns, validate the legitimacy and normalcy of being worried in such a situation, and avoid condescension and victim blaming.	“That tends to be the comfortable spot that we speak from as public health professionals and scientists. We tend to speak from a standpoint of science. Whereas what people in the community need is to be listened to and empathized with; to feel like they’re being heard and their concerns are being addressed.” (HD3)
Re-establish and Build Trust	Take visible and transparent action to address the PFAS contamination. Communicate to the community about what is being done, and proactively follow-up and maintain long-term consistent bi-directional communication between communities and government. One-on-one personal interactions, if implemented effectively, can help establish trust.	“Really the only way we can counter (lack of trust with the government) is through actions and continued communication … There are people in our state who definitely still do not trust us. One of the best things you can do as government agencies is just to continue to be in contact with the community, to continue to show them that we’re engaged, and that we’re here and we’re doing everything we can.” (HD1)
Provide Information and Actionable Guidance	Utilize a broad and inclusive approach to provide educational information about PFAS (e.g., background information, health risks, interpreting scientific information) and actionable guidance (e.g., ways to reduce exposure) that is straightforward and considers language barriers and the needs of low-income and “hard-to-reach” groups.	“Government should be honest and realistic when speaking with the community. Do not try to mitigate fear, but instead be upfront about what you don’t know and what you can’t officially say, but then offer practical and realistic guidance that is based in caution around chemical exposure, rather than leaning on the fact that ‘health impacts aren’t yet proven’.” (CM3)
Discuss Stress Carefully	Discussing psychosocial stress with community members experiencing PFAS contamination can easily be perceived as victim blaming or minimizing their concerns. Establish trust first, and then bring up psychosocial stress in the context of other health risks related to PFAS.	“We have someone in public health who deals with community stress and anxiety … We brought that person early on to a community meeting and had her try and speak directly to the stress and anxiety people felt. The feedback we heard from that engagement was negative. The community felt talked down to and that their concerns were being minimized.” (HD3)
Support Stress-Coping Activities	Help community members cope with stress by helping them take health-protective action and fostering social capital. Assist communities’ members to not only take personal action, but also be truly involved in decision-making related to the governmental PFAS response (e.g., a community advisory board). Additionally, assist, as possible (and as requested by the community), in the creation of grassroots community groups responding to PFAS contamination. Low/no cost counseling services may also be of need to some community members.	“The stories you hear from people across the country … who lost people, are struggling with harm, and are worried about their children moving forward are appalling. But then you look at that person and see how strong they are, they decided to take that energy and do something about it. Those people are all over the country and they are supporting people in their communities and people are seeing them. In a weird way it’s giving people hope … People are feeling validated that they’re counted and that they are seen, and that validation is very important to victims.” (CM2)
Build Capacity to Address Stress	Provide training/expertise and guidance for engaging with concerned communities, risk communication, integrating mental health counseling into public health response (if desired by the community), education/guidance for health care providers, and federal guidance to coordinate PFAS response across states.	“We had a training here called ‘emotions, outrage and public participation.’ So it was about how to work with communities that are angry. My biggest takeaway from that was people are angry, it’s not directed at you personally. It’s the situation that they’re frustrated with. Be empathetic, listen. So that’s my first piece of advice.” (HD2)
